# Impact of Hormones and Lifestyle on Oral Health During Pregnancy: A Prospective Observational Regression-Based Study

**DOI:** 10.3390/medicina60111773

**Published:** 2024-10-30

**Authors:** Liliana Sachelarie, Ait el haj Iman, Murvai Violeta Romina, Anca Huniadi, Loredana Liliana Hurjui

**Affiliations:** 1 Preclinics Department, Faculty of Medicine, Apollonia University, 700511 Iasi, Romania; 2 Preclinics Department, Faculty of Medicine and Pharmacy, University of Oradea, 410073 Oradea, Romania; athj.iman@gmail.com (A.e.h.I.); rominna.cuc@gmail.com (M.V.R.); 3 Clinics Department, Faculty of Medicine and Pharmacy, University of Oradea, 410073 Oradea, Romania; 4 Faculty of Medicine, “Grigore T. Popa” University of Medicine and Pharmacy, 700115 Iasi, Romania; loredana.hurjui@umfiasi.ro

**Keywords:** pregnancy, stomatognathic system (SS), hormonal changes, multiple regression, gingival inflammation (GI)

## Abstract

*Background and Objectives*: This study explores the impact of hormonal fluctuations during pregnancy and lifestyle factors on stomatognathic system (SS) health. The aim is to determine how pregnancy-related hormonal changes and oral hygiene behaviors affect the onset of stomatognathic issues, such as gingival inflammation (GI) and dental erosion (DE). *Materials and Methods:* A prospective, observational study was conducted with 100 pregnant women, divided into two groups: Group A (60 women with significant stomatognathic alterations) and Group B (40 women without such alterations). Multiple regression analysis was used to evaluate the influence of hormonal levels, oral hygiene habits, and vomiting episodes on stomatognathic health. *Results:* Age and socioeconomic status showed no significant association with stomatognathic health (*p* > 0.05). In contrast, elevated levels of estrogen (*p* = 0.001) and progesterone (*p* = 0.003) were significantly linked to the severity of stomatognathic changes. Oral hygiene habits also had a statistically significant impact (*p* = 0.02), while vomiting frequency was not an important factor (*p* > 0.05). *Conclusions:* Hormonal changes during pregnancy, particularly increased estrogen and progesterone levels, are key predictors of stomatognathic health. These findings suggest that while oral hygiene is important, hormonal fluctuations play a dominant role in influencing stomatognathic system (SS) health during pregnancy.

## 1. Introduction

The impact of pregnancy on systemic and oral health has been a subject of growing interest in recent years. Hormonal fluctuations, particularly increases in estrogen and progesterone levels, have been shown to influence various physiological changes in pregnant women, including alterations in the stomatognathic system (SS). The stomatognathic system (SS), which includes the teeth, jaws, and associated structures, can be affected by these hormonal changes, leading to conditions such as gingivitis, periodontitis, and dental erosion.

Pregnancy gingivitis, characterized by inflammation and bleeding of the gums, is one of the most common oral health issues during pregnancy. It affects nearly 60–70% of pregnant women, and its onset has been strongly linked to increased levels of estrogen and progesterone [1,2]. These hormones promote vascular changes and modify the immune response in gingival tissues, making them more susceptible to bacterial plaque accumulation [2]. Additionally, pregnancy-associated periodontitis, a more severe form of gum disease, has been associated with adverse pregnancy outcomes such as preterm birth and low birth weight [3,4].

Apart from soft tissue changes, dental erosion has been observed in some pregnant women, often due to the increased frequency of vomiting caused by morning sickness or hyperemesis gravidarum. This results in exposure of the teeth to stomach acid, leading to the demineralization of enamel [5]. Moreover, decreased salivary flow during pregnancy can exacerbate this process by reducing the natural buffering capacity of saliva [6].

The connection between hormonal changes and oral microbial shifts has also been studied. Increased progesterone levels may favor the growth of anaerobic bacteria, such as Porphyromonas gingivalis and Prevotella intermedia, which are known to play a role in periodontal disease [7,8]. These microbial changes, poor oral hygiene, and hormonal influences create a conducive environment for oral health deterioration during pregnancy.

While several studies have focused on the general relationship between pregnancy and oral health, there is still a gap in understanding how specific hormonal changes correlate with the severity of stomatognathic system (SS) alterations [9]. The current study aims to address this gap by exploring the association between hormonal fluctuations (particularly estrogen and progesterone) and the health of the stomatognathic system (SS) using a multiple regression approach. Understanding these correlations can aid in developing targeted interventions for improving oral health outcomes in pregnant women.

Higher levels of estrogen and progesterone lead to greater vascular permeability and capillary dilation within gingival tissues, which makes them more prone to inflammation. Specifically, estrogen promotes vascular permeability by altering the endothelial cells lining blood vessels, leading to fluid accumulation in the gingival tissues and increasing the risk of inflammation and bleeding. This intensified vascular response is a major contributing factor to pregnancy gingivitis, an oral condition affecting 60–70% of pregnant women [9].

Hormonal changes during pregnancy also influence the immune system, resulting in a modified immune response within gingival tissues. Progesterone can suppress local immune defense by reducing the effectiveness of neutrophils and macrophages against bacterial plaque. This immunosuppressive effect encourages bacterial accumulation and gingival inflammation, increasing the risk of periodontal disease progression during pregnancy [10].

Hormonal fluctuations alter the composition of the oral microbiota. Elevated progesterone, for example, supports the growth of specific anaerobic bacteria, such as *Porphyromonas gingivalis* and *Prevotella intermedia*, which are associated with periodontal disease. These bacteria thrive under the hormonal changes of pregnancy, intensifying inflammation and contributing to gingivitis and periodontal issues [9]. Additionally, dietary cravings and morning sickness-related vomiting can increase oral acidity, which in turn promotes enamel demineralization and raises the risk of dental caries [4].

Dental erosion during pregnancy is often exacerbated by frequent vomiting, especially in cases of hyperemesis gravidarum. The exposure of teeth to stomach acid demineralizes enamel, making the teeth more susceptible to caries. Additionally, reduced salivary flow, commonly reported during pregnancy, limits saliva’s natural buffering capacity, thereby increasing the risk of acid erosion and further compromising oral health [5].

## 2. Materials and Methods

### 2.1. Study Design

Over two years, this prospective, observational study was conducted at the Calla—Infertility Diagnostic and Treatment Center in Oradea, Romania. The study received approval from the Ethics Committee, under reference number 670 on 11 November 2021 to explore the correlation between hormonal fluctuations during pregnancy and stomatognathic system (SS) health. The study included 100 pregnant women, randomly selected from a clinical population. The participants were divided into two groups based on the presence or absence of significant stomatognathic alterations: Group A (n = 60) included women with GI, DC, and other stomatognathic issues; Group B (n = 40) included women without these stomatognathic alterations.

This study was conducted in compliance with the STROBE (Strengthening the Reporting of Observational Studies in Epidemiology) for observational studies and followed all recommended standards to ensure transparency and accuracy in methodology and findings. The STROBE element, including inclusion and exclusion criteria, definition and measurement of variables, and statistical analysis, were followed.

**Figure 1:**
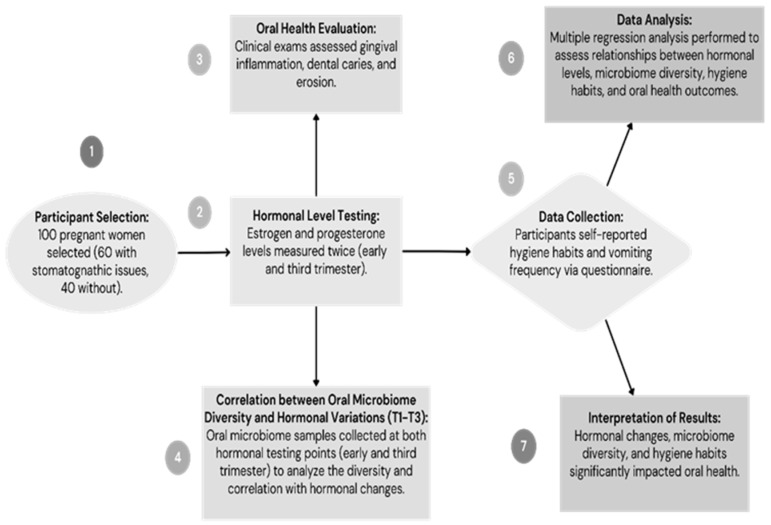
Workflow.

Inclusion criteria included pregnant women aged 18–40 in their second or third trimester who provided informed consent. Women with pre-existing systemic conditions, such as diabetes mellitus or autoimmune diseases, were excluded from the study (Figure 1).

The sample size for this study was established based on comparable studies in the literature that explored the relationship between oral health and hormonal factors during pregnancy. We included 100 participants, aligning with other research works that used sample sizes between 80 and 150 pregnant women to evaluate the effects of hormones and lifestyle on stomatognathic health. Laine (2002) assessed periodontal health in 124 pregnant women, showing that a sample of this magnitude is suitable for investigating hormonal effects on gingival and dental health during pregnancy [9]. Similarly, Figuero et al. (2010) conducted a study with 110 participants, examining immunological and clinical changes in the gums, thus offering valuable insights into the influence of hormonal fluctuations [10]. Additionally, Gürsoy et al. (2008) studied 102 pregnant women to assess periodontal changes, concluding that this sample size is sufficient for identifying significant associations between oral health and pregnancy-specific hormonal changes [1].

Over two years, this prospective, observational study was conducted at the Calla—Infertility Diagnostic and Treatment Center in Oradea, Romania. The study received approval from the Ethics Committee, under reference number 670 on 11 November 2021 to explore the correlation between hormonal fluctuations during pregnancy and SS health. The study included 100 pregnant women, randomly selected from a clinical population. The participants were divided into two groups based on the presence or absence of significant stomatognathic alterations: Group A (n = 60) included women with gingival inflammation (GI), dental caries (DC), and other stomatognathic issues; Group B (n = 40) included women without these stomatognathic alterations.

Inclusion criteria included pregnant women aged 18–40 in their second or third trimester who provided informed consent. Women with pre-existing systemic conditions, such as diabetes mellitus or autoimmune diseases, were excluded from the study (Figure 1).

Hormone levels were measured through routine blood tests to determine concentrations of estrogen and progesterone.

Each participant was tested twice: once at the beginning and once in the middle of the third trimester of pregnancy. The health of the stomatognathic system (SS) was evaluated through a clinical oral examination, focusing on GI, DC, and other signs of stomatognathic alteration. Oral health parameters were recorded by a dentist who specialized in dental care for pregnant women. Oral hygiene habits and the frequency of vomiting episodes were self-reported through questionnaires. Oral hygiene was categorized based on the frequency of brushing and flossing, while vomiting frequency was assessed based on the number of weekly episodes reported.

### 2.2. Statistical Analysis

Data were analyzed using SPSS version 27.0 (Chicago, IL, USA). Descriptive statistics were applied to characterize the study population. A multiple regression analysis was performed to investigate the relationships between independent variables (estrogen, progesterone, oral hygiene behaviors) and dependent variables (gingival inflammation (GI), periodontal pocket depth, dental erosion). Significance levels were set at *p* < 0.05.

## 3. Results

### 3.1. Baseline Characteristics

The median age of participants in Group A (those with stomatognathic alterations) was 29.6 years, with an interquartile range (IQR) of 26–32 years. In Group B (those without stomatognathic alterations), the median age was 29.2 years, with an IQR of 26–31 years. The statistical analysis showed no significant difference between the two groups based on age, with a *p*-value of 0.7207, Table 1.

In Group A, 58.3% of participants were from urban areas, and 41.7% were from rural areas. In Group B, 55% of participants were from urban areas, and 45% were from rural areas. A slight but significant difference was observed between the groups regarding their environment of origin, with a *p*-value of 0.0396.

In Group A, 66.7% of participants were in their second trimester, while 33.3% were in their third trimester. In Group B, 70% of participants were in their second trimester, and 30% were in their third trimester. The statistical significance for the trimester distribution was borderline, with a *p*-value of 0.05, indicating a nearly significant difference between the groups.

### 3.2. Hormonal Levels and Stomatognathic Health

Table 2 presents the relationship between hormonal fluctuations (specifically estrogen and progesterone) and gingival inflammation (GI) and the number of dental caries (DC). The estimated values in the table reflect the combined analysis of both groups, focusing on how variations in hormonal levels (increased estrogen and progesterone) correlate with stomatognathic health outcomes like GI and DC.

For participants with increased estrogen levels, the average GI score was 2.5 ± 0.3, indicating a moderate degree of inflammation.

Similarly, for those with increased progesterone levels, the average gingival inflammation (GI) score was slightly lower, at 2.3 ± 0.4.

Both hormone levels significantly impacted GI, as evidenced by the *p*-values (estrogen: 0.001, progesterone: 0.003), suggesting a strong correlation between higher hormone levels and increased GI.

In the estrogen group, 70% of participants experienced bleeding upon probing, a clinical marker for GI.

In the progesterone group, the percentage was slightly lower, at 65%. These percentages support the significant role that hormonal fluctuations play in exacerbating gingival inflammation (GI) during pregnancy.

The average number of DC observed in participants with increased estrogen levels was 4.1 ± 1.2, while for those with higher progesterone levels, the average number of caries was slightly lower, at 3.8 ± 1.4.

The correlation between hormone levels and the occurrence of DC was also statistically significant, with *p*-values of 0.002 for estrogen and 0.004 for progesterone, indicating that higher hormone levels are associated with a greater number of caries.

The *p*-values for both GI and DC concerning hormone levels are all below 0.05, meaning the relationships observed between hormonal levels (both estrogen and progesterone) and these stomatognathic health outcomes are statistically significant.

### 3.3. Oral Hygiene Habits

Oral hygiene behaviors, such as the frequency of tooth brushing and flossing, were statistically significant factors in the presence of stomatognathic alterations (*p* = 0.02). Women with poor oral hygiene habits exhibited more pronounced gingival inflammation (GI) and dental caries (DC), indicating a clear relationship between hygiene practices and oral health during pregnancy (Table 3).

### 3.4. Frequency of Vomiting Episodes

The frequency of vomiting episodes, although hypothesized to have an impact on stomatognathic health, did not show a statistically significant association with dental caries (DC) or gingival inflammation (GI) (*p* > 0.05). This suggests that hormonal changes, rather than vomiting-related acid exposure, were the primary contributors to the observed alterations.

Table 4 explores the relationship between the frequency of vomiting episodes and key stomatognathic health indicators, including gingival inflammation (GI) and dental caries (DC). The data show that the frequency of vomiting did not have a statistically significant association with these oral health issues, as indicated by *p*-values greater than 0.05.

Participants were categorized into three groups based on the frequency of vomiting episodes: 1–2 episodes, 3–4 episodes, and 5+ episodes per week. Across all vomiting frequency groups, the gingival inflammation (GI) index remained stable, with scores ranging from 2.2 to 2.4, reflecting moderate gingival inflammation (GI). The lack of statistically significant differences (*p* > 0.05) suggests that vomiting frequency was not a key driver of gingival inflammation (GI).

The percentage of participants experiencing bleeding on probing was consistent across vomiting frequency groups, with values ranging from 62% to 65. So, the frequency of vomiting had little impact on gingival bleeding. Similarly, the number of dental caries (DC) showed only minor variations between the vomiting groups, with averages ranging from 3.9 to 4.1. No significant relationship was found between vomiting frequency and the occurrence of dental caries (DC), as indicated by *p*-values above 0.05.

Both gingival inflammation (GI) and dental caries (DC) have *p*-values greater than 0.05, indicating that the frequency of vomiting episodes did not significantly impact stomatognathic health in the study group.

### 3.5. Correlation Between Oral Microbiome Diversity and Hormonal Variations (T1–T3)

The table below provides a detailed analysis of the correlation between oral microbiome diversity shifts from the first trimester (T1) to the third trimester (T3) and hormonal fluctuations (estrogen and progesterone levels).

Table 5 demonstrates the relationship between changes in the oral microbiome and hormonal fluctuations during pregnancy. Increased levels of estrogen and progesterone in the third trimester (T3) are associated with heightened gingival inflammation (GI) and an increased number of dental caries (DC).

The *p*-values indicate a significant correlation between hormonal changes and these oral health indicators, particularly in the case of estrogen (*p* = 0.001 for inflammation and *p* = 0.002 for caries), as well as progesterone (*p* = 0.003 for inflammation and *p* = 0.004 for caries). The findings suggest a stronger influence of hormonal fluctuations in the third trimester on oral health outcomes.

### 3.6. Regression Model

The multiple regression analysis revealed that estrogen and progesterone levels were significant predictors of stomatognathic health outcomes, accounting for 45% of the variance in the severity of gingival inflammation (GI) and dental caries (DC) (adjusted R^2^ = 0.45). Oral hygiene habits further contributed to the model’s predictive accuracy, while vomiting frequency had no significant effect.

Table 6 shows how various predictors (hormonal levels, oral hygiene habits, and vomiting frequency) influence stomatognathic health outcomes, such as GI and DC. The combined effect of both estrogen and progesterone levels explains 45% of the differences in GI and DC among the study participants and indicates that these two hormonal factors together have a significant role in determining the severity of these oral health issues during pregnancy. While oral hygiene may not be as strong a predictor as estrogen and progesterone, adding it to the model enhances the overall accuracy by providing additional relevant information that improves the model’s ability to explain the variation in gingival GI and DC outcomes.

Increased estrogen levels were found to be a significant predictor of increased GI, accounting for a substantial part of the variance (adjusted R^2^ = 0.45) in inflammation severity. This means estrogen levels strongly influence how severe the GI becomes during pregnancy. Higher estrogen levels were also linked to a significant increase in the number of DC. This demonstrates that hormonal fluctuations in estrogen directly affect oral health during pregnancy, contributing to both inflammation and caries. The *p*-value (0.001) shows a highly significant relationship with stomatognathic health.

Like estrogen, increased progesterone levels were also a significant predictor of GI, further contributing to the 45% variance explained by the model. The higher the progesterone levels, the more severe the GI observed.

Progesterone was also found to significantly contribute to the increase in DC. The hormonal changes that occur during pregnancy appear to have a direct and measurable impact on both inflammation and caries formation; the *p*-value (0.003) confirms the role of progesterone in influencing oral health outcomes.

Poor oral hygiene habits, such as infrequent brushing and flossing, were associated with more severe GI. While oral hygiene was not as dominant a predictor as hormone levels, it still contributed to the model’s predictive accuracy, indicating that poor oral hygiene worsens inflammation. Proper oral hygiene practices are important for reducing the severity of caries, even when there are hormonal changes; the *p*-value (0.02) shows a significant but smaller contribution of oral hygiene compared to hormone levels.

Despite initial assumptions that vomiting frequency might impact oral health (due to acid exposure), the analysis showed that vomiting episodes had no significant effect on GI. Similarly, vomiting frequency had no significant impact on the number of DC, as confirmed by *p* > 0.05, indicating no statistically significant relationship between vomiting frequency and stomatognathic health outcomes.

The combined influence of estrogen and progesterone levels accounts for 45% of the variance (adjusted R^2^ = 0.45) in GI and DC. This means nearly half of the observed differences in stomatognathic health can be explained by hormonal fluctuations during pregnancy.

Oral hygiene habits also improved the accuracy of the model, and the vomiting frequency did not contribute significantly.

## 4. Discussion

The results of this study confirm the significant role of hormonal changes during pregnancy in influencing stomatognathic health, specifically regarding GI and DC. Hormonal fluctuations, particularly increased levels of estrogen and progesterone, were found to have a strong impact on the severity of these oral health issues, a finding consistent with previous literature [10].

The present study demonstrated that estrogen and progesterone levels account for 45% of the variance in GI and DC severity. This finding is aligned with previous research showing the impact of pregnancy hormones on oral health. Hormones such as estrogen are known to increase vascular permeability and enhance the inflammatory response in the gingival tissue, which may explain the higher prevalence of GI among pregnant women [11,12]. Similarly, progesterone has been implicated in reducing the effectiveness of the body’s immune response to oral pathogens, contributing to worsened periodontal conditions [12,13,14].

This study builds on the work of Kandan et al. (2011), who found that hormonal fluctuations during pregnancy are associated with increased gingival bleeding and pocket depth, particularly in the third trimester. Our findings, which showed significant correlations between estrogen and progesterone levels and GI, support these conclusions [3].

While hormonal changes were the primary predictors of stomatognathic alterations, oral hygiene behaviors also played a crucial role. Women with poor oral hygiene habits exhibited more pronounced GI and DC. This is consistent with studies such as that of Sedghi et al. who emphasized the importance of maintaining good oral hygiene practices during pregnancy to mitigate the effects of hormonal fluctuations [15]. Our findings further support the assertion that oral hygiene habits can modify the risk of severe stomatognathic alterations, even in the presence of hormonal changes.

Importantly, the combination of increased hormone levels and poor oral hygiene behaviors exacerbates the severity of GI and DC. Some studies on pregnant women who maintain regular oral hygiene practices, such as daily brushing and flossing, show that they have significantly lower levels of gingival bleeding and caries [16,17,18,19]. This finding reinforces the importance of integrating oral health education into prenatal care programs.

So, this study found no statistically significant relationship between the frequency of vomiting episodes and oral health outcomes, including GI and DC. This contradicts earlier hypotheses that vomiting-induced acid exposure might contribute to dental erosion and caries. Similar findings observed that while nausea and vomiting are common in pregnancy, they are not significantly associated with increased oral health deterioration when controlled for other factors like oral hygiene and hormonal levels [20,21,22,23]. Hormonal changes may have a more direct and potent impact on oral health compared to acid exposure from vomiting.

The findings from this study have important clinical implications. Given the strong correlation between pregnancy hormones and stomatognathic health, it is essential for dental professionals to closely monitor the oral health of pregnant women, particularly those showing increased estrogen and progesterone levels. Proactive periodontal care during pregnancy can reduce the risk of GI and caries, especially in women who may have poor oral hygiene practices. Integrating dental check-ups into routine prenatal care could help in the early identification and management of pregnancy-related oral health issues [24,25,26]. Additionally, health professionals should emphasize the importance of maintaining good oral hygiene practices, especially for women in their second and third trimesters, when hormone levels are at their peak. Oral health education should be provided as part of prenatal care, and regular dental visits should be encouraged to mitigate the adverse effects of hormonal changes on the SS [1,27,28,29].

Recent studies have shown that hormonal changes during pregnancy affect local immunity and predispose individuals to gingival and periodontal conditions, highlighting the combined impact of estrogen and progesterone on gingival tissue (Romandini et al., 2020) [12]. Pregnancy-associated shifts in the oral microbiota have been observed, with an increase in anaerobic bacterial species that contribute to gingival inflammation and elevate the risk of caries (Lin et al., 2018) [14]. Additionally, there is a significant link between periodontal disease and adverse pregnancy outcomes, such as preterm birth and low birth weight, underscoring the need for preventive oral care during pregnancy (Bobetsis et al., 2020) [26].

This study has some limitations. The sample size, although sufficient for the scope of this research, could be expanded to include a more diverse population of pregnant women. Additionally, future studies should explore the long-term effects of pregnancy-induced hormonal changes on oral health, particularly postpartum.

Further research is also needed to determine whether personalized oral hygiene routines can reduce the impact of hormonal fluctuations on stomatognathic health. Research should be on longitudinal studies to track oral health changes throughout pregnancy and postpartum, allowing for a deeper understanding of how hormonal fluctuations impact stomatognathic health over time.

Additionally, factors such as nutritional status, stress levels, and specific hormonal markers beyond estrogen and progesterone could provide a more comprehensive view of the influences on oral health during pregnancy. Testing targeted preventive interventions, such as enhanced oral hygiene protocols or prenatal dental education programs, would also be valuable to determine effective strategies for minimizing pregnancy-related oral health issues. Such research can ultimately inform more tailored and preventive approaches within prenatal care.

## 5. Conclusions

This study provides a unique contribution by specifically analyzing the impact of hormonal levels on oral health during pregnancy using a regression model. The study shows that hormonal changes during pregnancy, especially estrogen and progesterone levels, significantly influence stomatognathic health GI and tooth decay. Oral hygiene has a supportive role, and dental care integrated into prenatal programs can prevent the deterioration of oral health in pregnant women.

## Figures and Tables

**Table 1:** Baseline characteristics.

Characteristic	Group A (n = 60)	Group B (n = 40)	*p*-Value
Age (median, IQR)	29.6 (26–32)	29.2 (26–31)	0.7207
Environment of origin (Urban/Rural)	35 Urban (58.3%)/25 Rural (41.7%)	22 Urban (55%)/18 Rural (45%)	0.0396
Pregnancy Trimester (II/III)	40 Second Trimester (66.7%)/20 Third Trimester (33.3%)	28 Second Trimester (70%)/12 Third Trimester (30%)	0.05
Socioeconomic status (Median ± SD)	Medium: 60%/High: 40%	Medium: 55%/High: 45%	0.081
Educational level	High school: 40%/University: 60%	High school: 42%/University: 58%	0.07

**Table 2:** Hormonal levels and stomatognathic health.

Hormonal Level	Estrogen Level (pg/mL)	Progesterone Level (ng/mL)	Gingival Inflammation (GI) Index (Mean ± SD)	Bleeding on Probing (%)	Number of Dental Caries (DC) (Mean ± SD)	*p*-Value Inflammation	*p*-Value Caries
Increased Estrogen	250 ± 30	15 ± 3	2.5 ± 0.3	70%	4.1 ± 1.2	0.001	0.002
Increased Progesterone	220 ± 25	20 ± 4	2.3 ± 0.4	65%	3.8 ± 1.4	0.003	0.004

**Table 3:** Oral hygiene habits and stomatognathic health.

Oral Hygiene Habit	Gingival Inflammation (GI) Index (Mean ± SD)	Bleeding on Probing (%)	Number of Dental Caries (DC) (Mean ± SD)	*p*-Value Inflammation	*p*-Value Caries
Brushing Frequency (Daily)	2.8 ± 0.4 (poor hygiene)	75% (poor hygiene)	4.5 ± 1.3 (poor hygiene)	0.02	0.02
Flossing Frequency (Weekly)	2.5 ± 0.3 (poor hygiene)	68% (poor hygiene)	4.0 ± 1.2 (poor hygiene)	0.02	0.02

**Table 4:** Frequency of vomiting episodes and stomatognathic health.

Vomiting Frequency (per Week)	Gingival Inflammation (GI) Index (Mean ± SD)	Bleeding on Probing (%)	Number of Dental Caries (DC) (Mean ± SD)	*p*-Value (Inflammation)	*p*-Value (Caries)
1–2 episodes	2.2 ± 0.5	62%	3.9 ± 1.0	>0.05	>0.05
3–4 episodes	2.3 ± 0.6	64%	4.0 ± 1.1	>0.05	>0.05
5+ episodes	2.4 ± 0.5	65%	4.1 ± 1.3	>0.05	>0.05

**Table 5:** Correlation between oral microbiome diversity and hormonal variations (T1–T3).

Trimester	Gingival Inflammation (GI) Index (Mean ± SD)	Bleeding on Probing (%)	Number of Dental Caries (DC) (Mean ± SD)	*p*-Value (Inflammation)	*p*-Value (Caries)
T1	2.0 ± 0.4	65%	3.2 ± 1.0	-	-
T3	2.6 ± 0.5	70%	4.1 ± 1.3	0.001 (Estrogen)	0.002 (Estrogen)
T3	2.4 ± 0.5	68%	3.8 ± 1.2	0.003 (Progesterone)	0.004 (Progesterone)

**Table 6:** Multiple regression analysis of stomatognathic health predictors.

Predictor	Effect on Gingival Inflammation (GI)	Effect on Dental Caries (DC)	*p*-Value	Adjusted R^2^
Estrogen Levels	Significant predictor; accounts for 45% of the variance in inflammation severity	Significant predictor; higher levels linked to more caries	0.001	0.45 (combined influence)
Progesterone Levels	Significant predictor; contributes to increased inflammation	Significant predictor; higher levels associated with more caries	0.003	0.45 (combined influence)
Oral Hygiene Habits	Contributes to the model’s accuracy; poor habits worsen inflammation	Contributes to increased caries; poor habits lead to worse outcomes	0.02	Improves model accuracy
Vomiting Frequency	No significant effect on inflammation	No significant effect on caries	>0.05	No effect

## Data Availability

The original contributions presented in the study are included in the article, further inquiries can be directed to the corresponding author/s.
